# Multidisciplinary lifestyle interventions for neurological disorders during the Silent phase (MINDS) study: a multi-omics randomized controlled trial protocol

**DOI:** 10.1186/s42466-024-00334-3

**Published:** 2024-08-01

**Authors:** Sara Taylor, Seerat Sachdeva, Sandra Darling, Kayela Arrotta, Lisa Gallagher, Alexis Supan, Gabrielle Shipta, Jim Perko, Judi Bar, Joe James, Iris Petschek, Anthony Lioi, Suman Kundu, Lisa Ellison, Lynn M. Bekris, Belinda Willard, Naseer Sangwan, Ignacio Mata, Hubert Fernandez, Irene Katzan, Devon Conway, Jagan Pillai, James Leverenz, Robyn M. Busch, Darlene Floden, Robert Saper, John Barnard, Andre Machado, Imad Najm, Vineet Punia

**Affiliations:** 1 Epilepsy Center, Neurological Institute, Cleveland Clinic, Cleveland, OH USA; 2 Dr. Sampurnanand Medical College, Jodhpur, Rajasthan India; 3 Primary Care Institute, Wellness and Preventive Medicine, Cleveland Clinic, Cleveland, OH USA; 4 Arts & Medicine Department, Patient Experience, Cleveland Clinic, Cleveland, OH USA; 5 Lerner Research Institute, Genomic Medicine Institute, Cleveland Clinic, Cleveland, OH USA; 6 Center for Neurological Restoration, Neurological Institute, Cleveland Clinic, Cleveland, OH USA; 7 Cerebrovascular Center, Neurological Institute, Cleveland Clinic, Cleveland, OH USA; 8 Mellen Center, Neurological Institute, Cleveland Clinic, Cleveland, OH USA; 9 Neurological Institute, Cleveland Clinic Lou Ruvo Center for Brain Health, Cleveland Clinic, Cleveland, OH USA; 10 Department of Quantitative Health Sciences, Cleveland Clinic, Cleveland, OH USA; 11 Department of Neurosurgery, Neurological Institute, Cleveland Clinic, Cleveland, OH USA; 12 Biomedical Engineering, Lerner Research Institute, Cleveland Clinic, Cleveland, OH USA

**Keywords:** Stroke, Epilepsy, Parkinson’s Disease, Alzheimer’s disease, Lifestyle interventions, Biomarkers, Randomized controlled trial

## Abstract

**Introduction:**

Given the prevalence and staggering cost of neurological disorders, there is dire need for effective early detection and intervention tools. Emerging evidence suggests that multidisciplinary lifestyle interventions (MLI) may mitigate the risk and progression of neurological disorders. The objectives of this protocol are (1) to test the impact of MLI on the progression of neurological disorders and (2) to identify multi-omic biomarkers for early stages of neurological disease and the impact of MLIs on these biomarkers.

**Methods and analysis:**

We present the Multidisciplinary lifestyle Interventions for Neurological Disorders during the Silent phase (MINDS) protocol, a randomized controlled trial of MLI in neurologically healthy older adults (≥ 50 years old) exhibiting elevated risk for common neurological disorders: stroke, epilepsy, Parkinson’s Disease, or Alzheimer’s disease and related dementias. Participants will be randomly assigned to intervention (*n* = 100) or control (*n* = 100) groups. The intervention group will receive 3 months of weekly 2-hour sessions on diet education, yoga, music therapy, and cognitive skills training. The participants’ neurological health and engagement in relevant lifestyle practices will be assessed at regular intervals for 12 months. Neuroimaging and samples for multi-omic analyses will be collected at baseline, and at 3 months and 12 months after enrollment. Primary outcomes will be signs of progression of the neurological disorder risk that qualified them for study enrollment or a clinical diagnosis of the disorder. Secondary and exploratory outcomes will be based on self-reported health and multi-omic data. Data analysis will include between-group and longitudinal within-group analyses.

**Perspectives:**

The MINDS protocol and trial aims to clarify the impact of MLI on the progression of neurological disorder risk or diagnosis in older adults and to identify biomarkers that can be used to confirm MLI efficacy. The ability to validate the impact of MLI on neurological disorder progression based on biomarker data allows the identification of individuals most likely to benefit from such therapies in the early stages of neurological disease.

**Trial registration:**

The trial is registered on the National Institutes of Health (NIH) ClinicalTrials.gov (NCT05984056) site. It was registered on August 2nd, 2023. The trial has full approval of the Cleveland Clinic Internal Review Board.

**Supplementary Information:**

The online version contains supplementary material available at 10.1186/s42466-024-00334-3.

## Background

In 2017, approximately 12 million people in the United States had at least one of four major neurological disorders: Parkinson’s Disease (PD), Alzheimer’s disease and related dementias (ADRD), epilepsy, and stroke [1]. Globally, these neurological disorders of older adults were estimated to be responsible for over 8.5 million deaths and the loss of nearly 190 million disability-adjusted life years in 2019, with increases likely given the rapidly aging population and longer life spans [2]. 

The development of preventive measures and early intervention strategies to reduce this disease burden of neurological disorders is needed, as candidates for early intervention can be identified based on prodromal features, biomarkers, and clinical risk factors. Lifestyle interventions, such as yoga, music therapy, cognitive skill, dietary interventions, reductions in alcohol and tobacco use, and exercise, can impact the risk of development and progression of neurological disorders in older adults. These lifestyle interventions can alleviate the impact of PD symptoms on quality of life, have positive effects on behavioral, cognitive, and psychological symptoms in ADRD, impact cognitive decline in the pre-dementia stages, lower blood pressure, and prevent/treat hypertension [3–10]. Together, results indicate clear benefits of lifestyle interventions on neurological disorder progression. Evidence also demonstrates that multidisciplinary lifestyle intervention (MLI) has greater impact on neurological disorder progression compared to a single lifestyle change [11]. However, the mitigation of risk of development and progression of neurological disorders in older adults through MLI is challenged by the lack of reliable biomarkers that can predict the influence of MLI on preclinical indicators disease and the progression to disease state.

Here, we present the Multidisciplinary lifestyle Interventions for Neurological Disorders during the Silent phase (MINDS) study protocol for an open-label, randomized controlled trial to investigate the impact of MLI on neurological disorder progression in older adults exhibiting elevated risk of neurological disease, and to identify biomarkers that can be used to confirm MLI efficacy.

## Methods

### Aims of the trial

We aim to test the impact of MLI on neurological disorder progression in patients identified as at risk of stroke, epilepsy, PD, or ADRD associated with the a) transition from ‘at-risk’ preclinical (silent) stage to the worsening of clinicopathological changes or a diagnosis of a neurological disorder and b) the progression of pathological changes leading to the development of a neurological disorder. An additional goal of the MINDS study is to identify biomarkers that predict MLI’s efficacy in disease prevention or neuropathology progression in these patients.

### Study description and study design

**Fig. 1:**
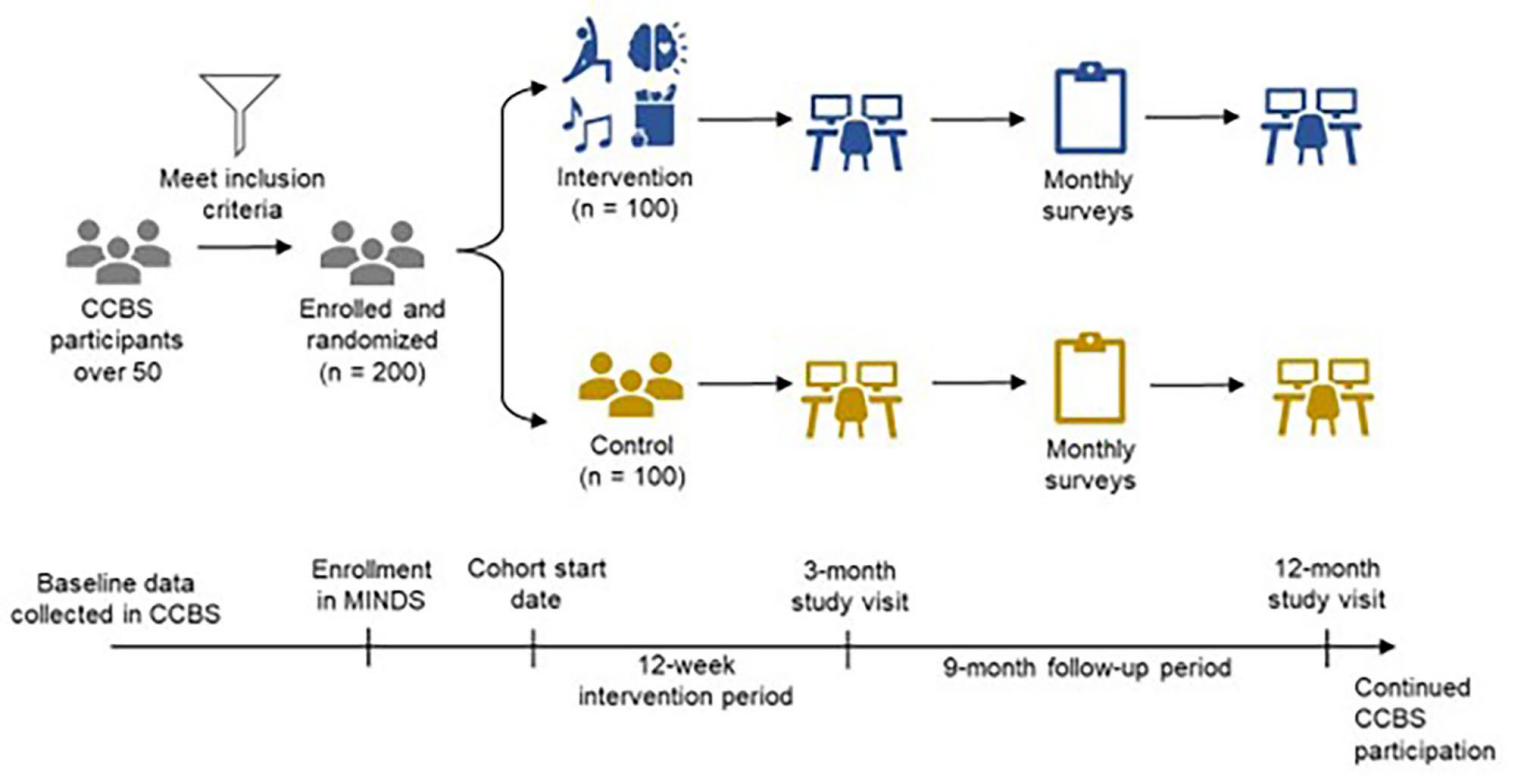
Study Timeline. MINDS study timeline, including recruitment, randomization, and data collection, shown from participant selection through the final study visit CCBS = Cleveland Clinic Brain Study. MINDS = Multidisciplinary lifestyle Interventions for Neurological Disorders during the Silent phase

This 12-month study is an open-label randomized controlled trial with parallel participant assignment into intervention and control arms (Fig. 1). The study will enroll individuals from the ongoing Cleveland Clinic Brain Study (CCBS), a longitudinal study of neurologically healthy adults (≥ 50 years old) at the Cleveland Clinic academic medical center, who are at-risk for transitioning to a neurological disorder [12]. A study team member who has been trained in the study protocol will provide the participant with information related to the study, obtain informed consent, and enroll the participant in the study if they choose to participate. CCBS participants undergo a thorough history and physical examination conducted by advanced practice providers with specialization in neurological care; a battery of surveys on quality of life, sleep, and mental health; neuropsychological testing [NP; includes the Brief Assessment of Cognitive Health (BACH), Tombaugh Trails test, Rey Auditory Verbal Learning Test (RAVLT), Boston Naming Test (BNT), Digit Span, Judgment of Line Orientation (JLO), and the Activities of Daily Living Questionnaire (ADLQ)]; brain magnetic resonance imaging (MRI); optical coherence tomography (OCT); and overnight electroencephalography (EEG) and polysomnography (PSG) along with biospecimen (blood, stool) collection for genomics, proteomics, and metabolomics analyses. Questionnaires include Perceived Stress Scale (PSS-10); Generalized Anxiety Disorder 7 Item (GAD-7); Patient Health Questionnaire Depression Scale (PHQ-8); Neuro-QoL (Quality of Life in Neurological Disorders) surveys for Sleep Disturbance, Ability to Participate in Social Roles and Activities, Lower Extremity Function, and Upper Extremity Function; PROMIS (Patient-Reported Outcomes Measurement Information System) Global Health, Healthy Aging Activity Engagement Scale, and General Self Efficacy Scale.This 12-month study is an open-label randomized controlled trial with parallel participant assignment into intervention and control arms (Fig. 1). The study will enroll individuals from the ongoing Cleveland Clinic Brain Study (CCBS), a longitudinal study of neurologically healthy adults (≥ 50 years old) at the Cleveland Clinic academic medical center, who are at-risk for transitioning to a neurological disorder [12]. A member of the study team who has been trained in the study protocol will provide the participant with information related to the study, obtain informed consent, and enroll the participant in the study if they choose to participate. CCBS participants undergo a thorough history and physical examination conducted by advanced practice providers with specialization in neurological care; a battery of surveys on quality of life, sleep, and mental health; neuropsychological testing [NP; includes the Brief Assessment of Cognitive Health (BACH), Tombaugh Trails test, Rey Auditory Verbal Learning Test (RAVLT), Boston Naming Test (BNT), Digit Span, Judgment of Line Orientation (JLO), and the Activities of Daily Living Questionnaire (ADLQ)]; brain magnetic resonance imaging (MRI); optical coherence tomography (OCT); and overnight electroencephalography (EEG) and polysomnography (PSG) along with biospecimen (blood, stool) collection for genomics, proteomics, and metabolomics analyses. Questionnaires include Perceived Stress Scale (PSS-10); Generalized Anxiety Disorder 7 Item (GAD-7); Patient Health Questionnaire Depression Scale (PHQ-8); Neuro-QoL (Quality of Life in Neurological Disorders) surveys for Sleep Disturbance, Ability to Participate in Social Roles and Activities, Lower Extremity Function, and Upper Extremity Function; PROMIS (Patient-Reported Outcomes Measurement Information System) Global Health, Healthy Aging Activity Engagement Scale, and General Self Efficacy Scale.

### Arms and interventions

Two hundred participants will be randomized equally to two study arms (*n* = 100 each): the intervention arm and the control observational arm. The sequence of group allocation was generated by the study data scientist using computer-generated random numbers and uploaded to the secure study REDCap database, where it can be administered by the study team. Each participant’s start date is based on participant availability, and, once enough participants (at least five) are identified that can meet at the same time, an intervention group and matching control group of comparable size will begin study activities on the same schedule. The experts completing quality assurance of the testing and analysis of the multi-omic data, including imaging, electrophysiology, and patient reported outcome measures are blinded to group allocation.

The intervention group will receive synchronous, virtual 2-hour weekly intervention sessions for 12 weeks. The control group will receive the standard of care during this time. Both groups will receive a MINDS study activity survey (see Supplemental Material 1) every 2 weeks during the first 12 weeks, followed by every month until the end of the 12-month study period. Each two-hour intervention session includes check-in and discussion of homework, followed by two 50-minute sessions, split evenly between two of the four intervention domains, with rotation through the domains and topics each week so that participants receive an even dose of intervention domain. See Supplemental Material 2 for an overview of the 12-week intervention sessions.

#### Yoga

During instructor-led, gentle yoga sessions, the yoga intervention focuses on practicing breathing, meditation, and mindfulness. A recent meta-analysis indicates that yoga leads to improved cognitive function acutely (i.e., right after a session), as well as chronically [13]. Yoga sessions in the MINDS study are led by certified yoga therapists.

#### Music therapy

In music therapy, participants are taught to use music to express themselves, reduce stress, and cope with daily challenges. Existing literature suggests music therapy benefits social, cognitive, motor, and emotional/psychological functioning in healthy older adults and patients with neurological disorders [4, 7, 14]. NIH has endorsed exploring the use of music therapy in brain disorders of aging [15]. Music therapy sessions are administered by a clinical music therapist.

#### Nutrition and culinary medicine

This educational intervention teaches cooking skills and how different foods can promote or inhibit healthy cognitive aging. ​The MINDS diet is similar to the MIND diet (i.e., encouraging plant-based foods and limiting foods concentrated with sugar or saturated fats), but emphasizes reducing consumption of red meat and other inflammatory foods, and adding Greek yogurt. Adherence to the MINDS diet will be tracked passively biweekly for the first 3 months and then monthly for the remainder of the study as part of the MINDS Study Activity Survey (see Table 1 for timing details). The survey results will be scored by one of the blinded investigators based on the MINDS diet daily checklist (Supplemental Material 3). A team of board-certified dieticians and American Culinary Federation certified chefs with years of experience in the healthcare industry will deliver these sessions.

**Table 1:** Study schedule of enrollment and assessments. Collected data elements and their timing listed at the time of enrollment, allocation to either intervention or control, and post-allocation. All post-allocation time-points are relative to the time of enrollment, while enrollment occurs before allocation (represented by -t). Grey shaded cells with a checkmark (✓) indicate data collected through Cleveland Clinic Brain Study. All other cells with an ‘X’ indicate data collected through the MINDS study. The attendance survey and post-intervention feedback survey are only collected for participants in the intervention arm

	STUDY PERIOD					
	Enrollment	Allocation	Post-Allocation			
Timepoint	-t	0	Months 1–3	Month 3	Months 4–12	Month 12
**Enrollment**						
Qualifying risk factor	✓					
Reading score (WRAT-4)	✓					
Eligibility Criteria	X					
Informed Consent	X					
Allocation		X				
**Assessments**						
Clinical History and Examination	✓			X		✓
Blood sample	✓			X		✓
Stool sample	✓			X		✓
NP Testing	✓			X		X
ECG	✓					✓
Polysomnography	✓					X
EEG	✓					X
MRI	✓					X
Smell Test (UPSIT)	✓					✓
OCT Scan	✓					X
Questionnaires	✓			X		✓
MDS-UPDRS	X			X		X
MINDS Study Activity Survey		X	*Bi-weekly*		*Monthly*	X
**Intervention Arm Only**						
Attendance survey			*Weekly*			
Post-intervention feedback survey				X		

#### Cognitive skills

This intervention teaches participants cognitive compensatory strategies and more general psychosocial strategies to optimize brain health. Brain health strategies include mental engagement, social engagement, exercise, and stress management. Cognitive compensatory strategies are intended to mediate and accommodate for any cognitive difficulties (such as impaired working memory). These strategies may increase functional independence, reduce the impact of cognitive changes related to aging, and delay the onset and progression of neurological disorders [16, 17]. Sessions include group counseling led by a licensed neuropsychologist.

### Outcome measures

The primary outcome of the MINDS study is the progression in the clinicopathological features specific to the neurological disorder for which the participant has a risk factor. Progression criteria for each disorder are described in Table 2. The secondary outcomes include the appearance of inclusion criteria (see Table 2) for neurological disorders other than the one that led to their study enrollment and self-report questionnaires in the following domains: quality of life in neurological disorders, depression symptoms, anxiety symptoms, self-efficacy, stress, and global health (see Table 3 for details). Exploratory outcomes for this study are neurological disease-related genomic, proteomic, metabolomic, and gut microbiome RNA biomarkers collected at 3 time points. The schedule for all outcome measurements and other assessments is reported in Table 1. Multi-omics data to be explored in the study includes the following:

**Table 2:** Inclusion and progression criteria by neurological disorder. Inclusion and progression criteria for each neurological disorder described

Neurological Disorder	Inclusion Criteria	Progression Criteria
Stroke	Moderate to severe white matter changes on MRI	Additional asymptomatic cortical infarct **Or** Further increase in white matter disease MRI changes **Or** Clinical stroke
Epilepsy	At least one epileptiform discharge (spike, polyspike, sharp wave) on overnight EEG	Increase in burden (per hour) of epileptiform discharges **Or** Electrographic seizure on EEG **Or** Clinical seizure
ADRDs	≤ 40 on subjective memory self-assessment on BACH **And** At least one memory task score (RAVLT Trial 7 or Total Score) to ≤ 1.5 SD below population mean	Increase in number of NP testing domain scores to ≤ 1.5 SD below population mean **Or** Drop in NP testing scores to ≤ 2SD below the mean **Or** Clinical diagnosis of dementia
Movement Disorders	Hyposmia (age and sex matched score ≤ 10th percentile) on the University of Pennsylvania Smell Identification Test (UPSIT)	> 4 points increase on modified MDS-UPDRS administered remotely **Or** Parkinson’s Disorder diagnosis **Or** Initiation of dopaminergic therapy

**Table 3:** Self-report questionnaires. Self-report questionnaires and phenotypic domains

Domain	Questionnaire Name
Stress	Perceived Stress Scale (PSS-10)
Generalized anxiety symptoms	Generalized Anxiety Disorder 7 Item (GAD-7)
Depression symptoms	Patient Health Questionnaire Depression Scale (PHQ-8)
Self-efficacy	General Self Efficacy Scale
Quality of life related to neurological disorders	Neuro-QoL - Sleep Disturbance
	Neuro-QoL - Ability to Participate in Social Roles and Activities
	Neuro-QoL - Lower Extremity Function
	Neuro-QoL - Upper Extremity Function
General health	PROMIS (Patient-Reported Outcomes Measurement Information System) Global Health
Healthy activities related to aging	Healthy Aging Activity Engagement Scale

#### Whole genome sequencing

will be used to score possible pathogenic variants for any neurological disorders based on ClinVar annotation and will extract the necessary genotypes to calculate polygenic risk scores (PRS) for each participant using publicly available Genome-Wide Association data for Parkinson’s Disease, Alzheimer’s Disease, Epilepsy, and Stroke.

#### Proteomics

Proteomic analyses will use O-Link NeurX 96 to analyze 92 exploratory and established protein markers of neurology-related diseases and biological processes. Neurofilament light polypeptide will be measured using the R-PLEX Human Neurofilament L Assay from Meso Scale Discovery (K1517XR, MSD; Rockville, MD). Protein assays for Aβ40, Aβ42, total tau, and pTau 181 will be performed using commercially available digital immunoassays (Neurology 3-Plex A Advantage Kit and pTau-181 Advantage V2.1 Assay Kit) on the Simoa SR-X platform (101,995 and 104,111, Quanterix; Billerica, MA) according to the manufacturer’s instructions.

#### Metabolomics

Sample metabolomes will be analyzed using an untargeted metabolomics approach involving metabolite extraction prior to analysis by high-performance liquid chromatography coupled to mass spectrometry (LC-MS). Resulting LC-MS data will be analyzed to determine the relative abundance of the observed metabolites across all samples and to identify metabolites.

#### Gut microbiome

We will use whole genome sequencing to analyze the gut microbiome’s taxonomic and functional dynamics by examining the microbial community’s complete (meta)transcriptome within the gut using MetaPhlAn4 and HUMAnN 3.

#### Sample size calculation

Our enrollment goal is 200 participants randomized to each group (intervention and control). Based on an attrition rate of 10%, we estimate we will have 180 participants with data for analysis. For primary outcomes analysis of binary outcomes (i.e., progression/no progression), the detection of a standard large effect size (odds ratio = 4.27, converted from Cohen’s *d* = 0.8) of the intervention at 0.8 power with a two-sided alpha of 0.05 would require 18% of the control group attaining progression. For secondary outcomes between group mixed-effects regression analysis, we will be able to detect a medium effect size (Cohen’s *f* = 0.27) at 0.8 power and a 5% significance level.

#### Data analysis plan

We will use a modified intention-to-treat population approach and include participants with valid baseline data and at least one post-baseline data point. Missing data among these participants will be handled using multiple imputations, clearly described, and with sensitivity analysis to test the robustness of the results.

Primary outcome analysis will be completed using a Fisher’s exact test to compare the proportions of progression in the intervention and treatment groups. To analyze secondary and exploratory outcomes between groups, we will use mixed-effects regression models to evaluate change in outcome as a function of group, time, and group-by-time interaction. We will also conduct longitudinal analysis within groups using repeated measures ANOVA to compare measures at time points, with covariates as fixed effects. Effect sizes and confidence intervals will be reported. Additionally, we will assess baseline sample characteristics and test for any baseline group differences using two-sample t-tests for continuous variables and Chi-square tests for categorical variables. We will also conduct an adherence analysis by reporting intervention session attendance rate and summary statistics for self-reported engagement with intervention domains and testing for any differences in these adherence metrics based on cohort number, age/sex, and other covariates. We will conduct sensitivity analyses aligned with best practices to see if the outcome analysis results are robust to any potential confounders, including baseline imbalance or intervention non-compliance.

For further exploratory analyses of relationships of multi-omic biomarkers with MLI, we will assess proteomic, metabolomic, and microbiomic biomarkers from our selected panels for detectable change over the course of the study, using a linear mixed-effects model, controlling for age, sex, and education, to determine whether biomarker concentrations change over time (baseline to 3 months and 12 months). For the biomarkers with significant time coefficient, we will re-run the model, adding a lifestyle score based on self-reported engagement with the intervention domains, to test for a role of lifestyle in the change over time. Additionally, we will test for simple associations between lifestyle and biomarkers by calculating Spearman correlations of each biomarker with self-reported engagement with each intervention domain, as well as with a composite total at baseline, 3 months, and 12 months. Finally, we will complete integrative multi-omic analysis that tests for covariance in changes in biomarkers over time.

### Eligibility criteria

CCBS participants with increased risk of developing ADRD, PD, stroke, or epilepsy are eligible for the MINDS study. Risk factors for development of these disorders are based on the current available evidence (Table 2). These factors are not diagnostic for ADRD, PD, stroke, or epilepsy. The four neurological disorders show significant clinicopathological overlap, and a MINDS-eligible participant enrolled for stroke risk factors may develop dementia before a clinical stroke, or a participant enrolled due to risk of a neurodegenerative disorder may later develop seizures before the clinical manifestation of dementia. Study enrollment began in July 2023 and is expected to finish in December 2024.

### Risk factor inclusion criteria

#### Parkinson’s disease

Most patients diagnosed with PD show hyposmia that predates diagnosis by years. We define hyposmia as a score at or below the 10th percentile based on age and sex on the University of Pennsylvania Smell Identification Test (UPSIT). We used the 10th percentile as the hyposmia cut-off to increase the specificity and yield for progression to PD.

#### Alzheimer’s disease and related dementias

Participants qualify as at-risk for dementia development with a combination of low semantic memory scores and poor subjective memory assessment, which show during the prodromal phase as early as two decades before clinical onset. Differences in amyloid-beta biomarkers and semantic memory tasks are early indicators of diagnosis. We will use semantic memory for inclusion of at-risk participants performing 1.5 or more standard deviations below the healthy population mean on the Rey Auditory Verbal Learning Test (RAVLT). To enrich the study population with high-risk individuals, we will assess subjective memory via the Brief Assessment of Cognitive Health (BACH), with a score ≤ 40 qualifying participants for inclusion.

#### Stroke

Severe age-related white matter changes predict stroke outcomes. CCBS structural MRIs are rated by a board-certified neuroradiologist using the Fazekas scale criteria: Grade 0 (“none”), Grade 1 (“mild”), Grade 2 (“moderate”), and Grade 3 (“severe”). Participants graded as moderate to severe white matter hyperintensities are considered at-risk for stroke and qualify for the MINDS study.

#### Epilepsy

The epilepsy inclusion criteria include select EEG features commonly seen in patients with epilepsy (i.e., interictal epileptiform discharges; IEDs). For the MINDS study, qualifying epileptiform discharges include spikes, sharp waves, and polyspikes. CCBS participants undergo an overnight (at least 6 h) EEG, which will be reviewed by a registered EEG technologist and neurologist with expertise in clinical EEG and epilepsy care. In addition, all potential participants undergo an independent review by a board-certified neurophysiologist and epileptologist for confirmation of IEDs as defined by the International Federation of Clinical Neurophysiology criteria (19).

#### Exclusion criteria

Participants with impaired ability to participate in the study’s interventions will be excluded. Participants actively engaged in two or more of the study intervention domains are not eligible for participation. Participants scoring below an 8th grade English reading level on the WRAT-IV Reading subset are also excluded. Finally, participants who require a legally authorized representative (LAR) or are not able to consent for themselves are not eligible for participation.

### Contacts (sponsors and collaborators, investigators)

The MINDS study was initiated at the Neurological Institute at Cleveland Clinic with the funding support of the Ohio Department of Higher Education. The lead PI is Dr. Vineet Punia, a neurologist specializing in epilepsy.

### Perspective

This study will benefit from the enrollment of deeply characterized neurologically healthy older adults (≥ 50 years) with risk factors for the four most common neurological disorders that share clinicopathological features. We will test the impact of multidisciplinary lifestyle interventions (MLI; diet, yoga, music and cognitive training) delivered using videoconferencing technology, which has the potential to be highly accessible and scalable. We will analyze the impact of this MLI on a variety of patient reported outcomes. The study’s ability to detect a large effect of MLI on the primary outcome of clinicopathological progression with sufficient power via group analysis depends on an incidence of progression (> 18%), which may be difficult to attain in the limited 12-month study period. This limitation will be mitigated by analysis of possible dose effects across groups via the MLI engagement data and supported by analysis more sensitive secondary outcomes. Additionally, the multimodal, multi-omics tests (neurophysiology, neuroimaging, genomics, proteomics, metabolomics, gut microbiome transcriptomics) will help explore biomarkers of early disease progression and MLI’s impact on them. This study aims to contribute to the body of work aiming to develop effective early interventions for neurological disease in older adults as well as explore patient reported outcomes and biomarker candidates for use in future trials.

### Electronic supplementary material

Below is the link to the electronic supplementary material.

Supplementary Material 1

## Data Availability

The sharing of anonymized participant-level data and statistical code from the study will be considered based on a reasonable request from a qualified investigator for rigorous scientific inquiry.

## Abbreviations

ADLQ: Activities of Daily Living Questionnaire
ADRD: Alzheimer’s disease and related dementias
ANOVA: Analysis of Variance
BACH: Brief Assessment of Cognitive Health
BNT: Boston Naming Test
CCBS: Cleveland Clinic Brain Study
ECG: Electrocardiogram
EEG: Electroencephalography
GAD-7: Generalized Anxiety Disorder 7 Item
IED: Interictal Epileptiform Discharge
IRB: Internal Review Board
JLO: Judgment of Line Orientation
LAR: Legally Authorized Representative
LC-MS: High-performance Liquid Chromatography Coupled to Mass Spectrometry
MDS-UPDRS: Movement Disorder Society – Unified Parkinson’s Disease Rating Scale
MIND: Mediterranean- Dietary Approaches to Stop Hypertension (DASH) Intervention for Neurodegenerative Delay diet
MINDS: Multidisciplinary Lifestyle Interventions for Neurological Disorders During the Silent Phase
MLI: Multidisciplinary Lifestyle Interventions
MRI: Magnetic Resonance Imaging
Neuro-QoL: Quality of Life in Neurological Disorders
NIH: National Institutes of Health
NP: Neuropsychological
OCT: Optical Coherence Tomography
PD: Parkinson’s disease
PEA: Proximity Extension Assay
PHQ-8: Patient Health Questionnaire Depression Scale
PROMIS: Patient-Reported Outcomes Measurement Information System (Global Health)
PRS: Polygenic Risk scores
PSG: Polysomnography
PSS-10: Perceived Stress Scale
RAVLT: Rey Auditory Verbal Learning Test
REDCap: Research Electronic Data Capture
SPIRIT: Standard Protocol Items: Recommendations for Interventional Trials
UPSIT: University of Pennsylvania Smell Identification Test
WGS: Whole Genome Sequencing
WRAT-IV: Wide Range Achievement Test- 4th Edition
