# Supplementary material for: Multidisciplinary lifestyle interventions for neurological disorders during the Silent phase (MINDS) study: a multi-omics randomized controlled trial protocol
Source: Neurol Res Pract. 2024 Aug 1;6:39. doi: 10.1186/s42466-024-00334-3 (PMC11293137; doi:10.1186/s42466-024-00334-3)
Supplement: Supplementary file 1 — Supplementary Material 1 [file 42466_2024_334_MOESM1_ESM.docx]

**Supplemental Material 1.** MINDS Study Activity Survey

General Instructions: Please complete the survey based on what you did in the last 7 days.


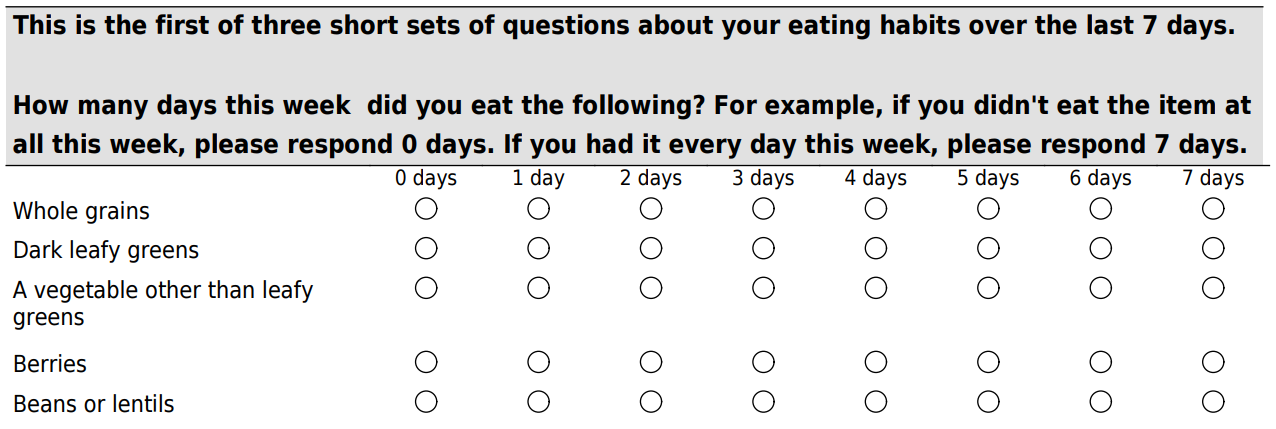


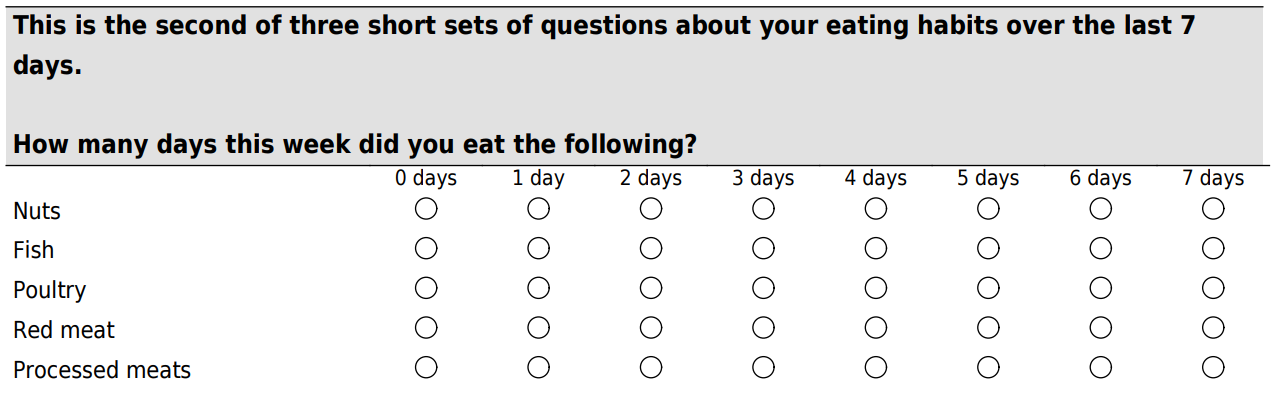


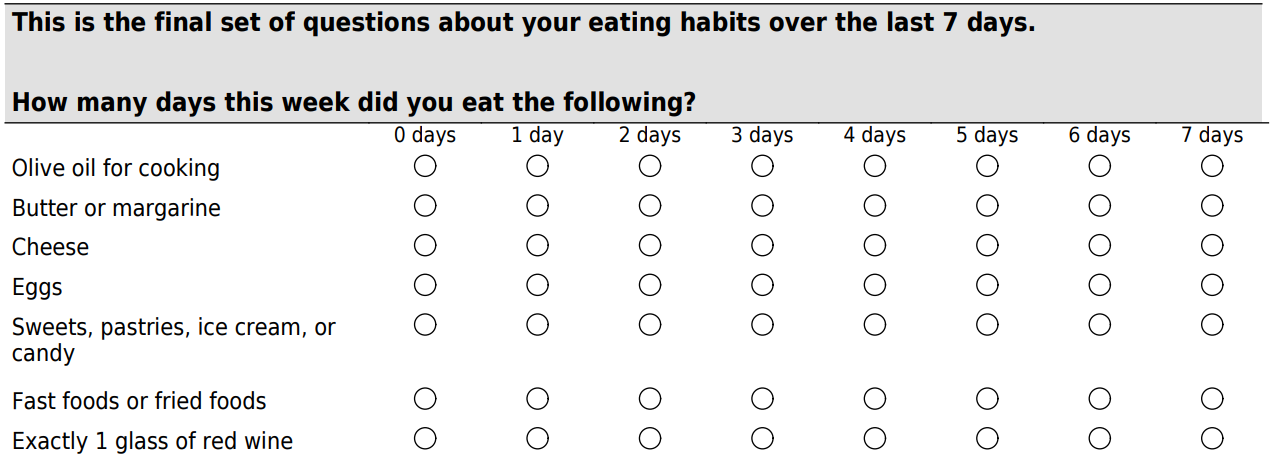


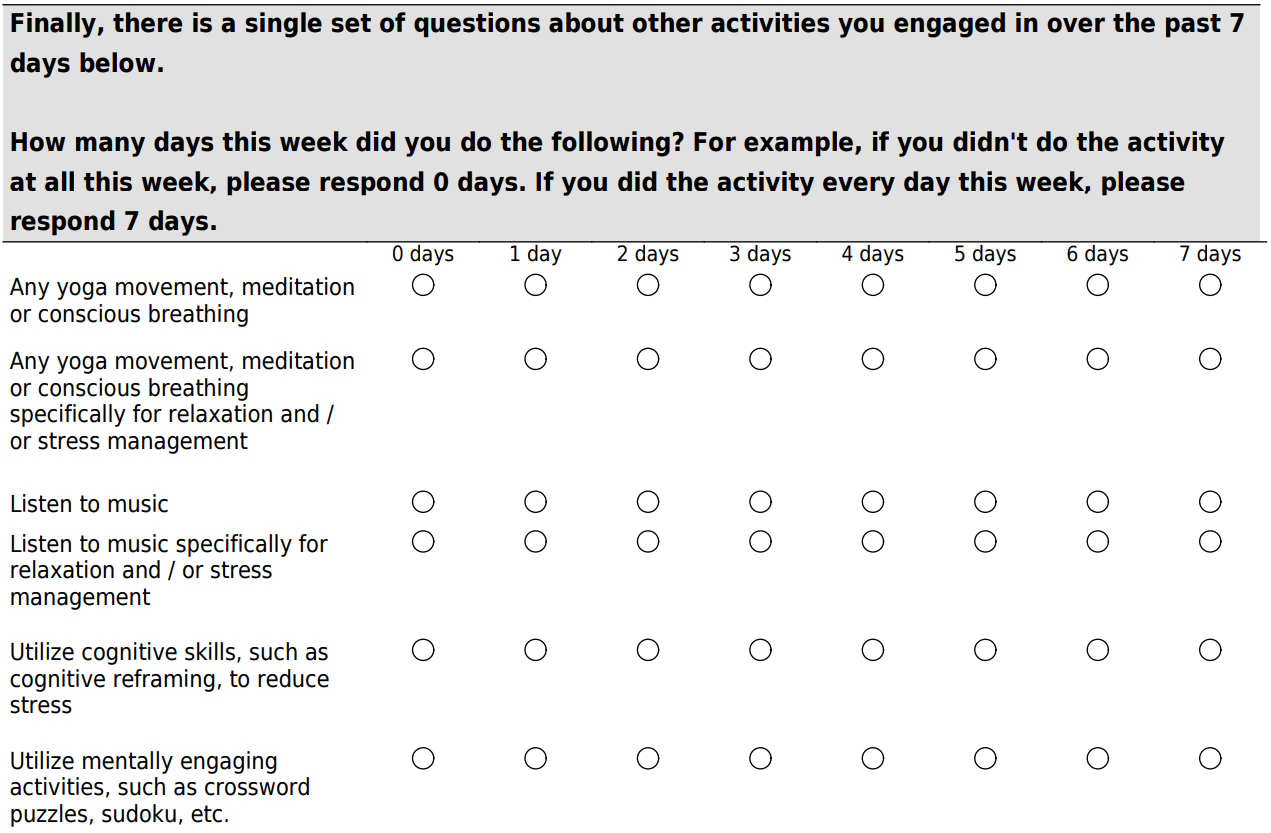


**Supplemental Material 2.** 12-weeks intervention sessions overview


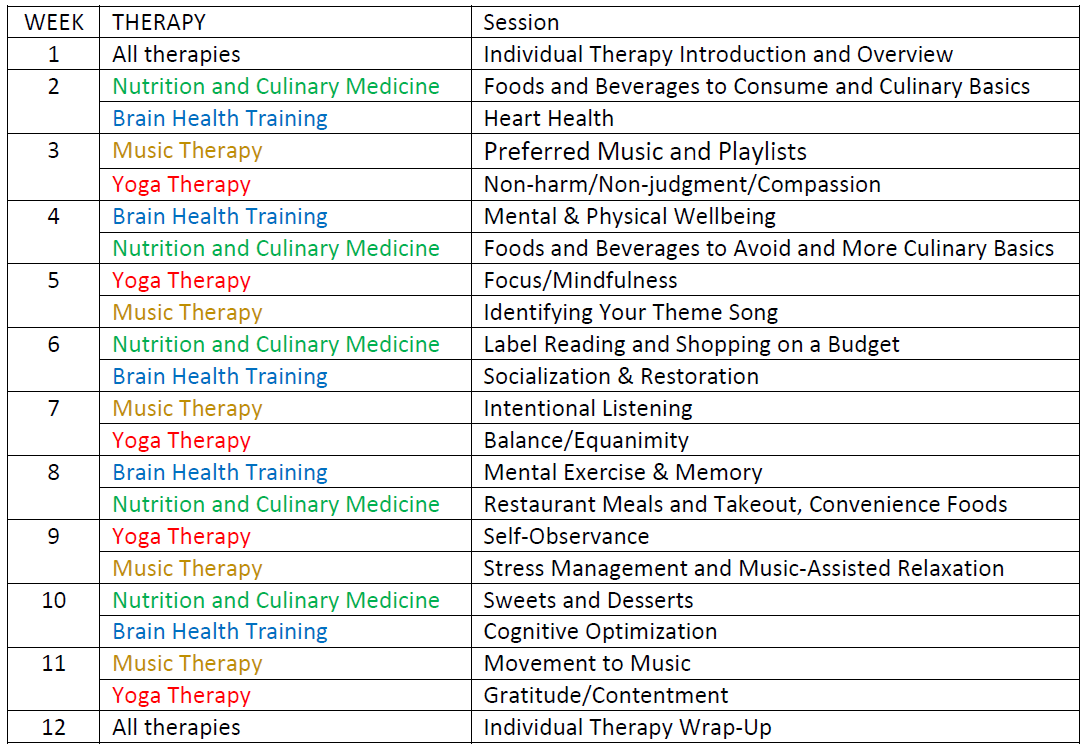


**Supplemental Material 3.** CC-MINDS diet daily checklist

| **Food group and quantity** | **Mon** | **Tue** | **Wed** | **Thu** | **Fri** | **Sat** | **Sun** | **Mon** | **Tue** | **Wed** | **Thu** | **Fri** | **Sat** | **Sun** |
| --- | --- | --- | --- | --- | --- | --- | --- | --- | --- | --- | --- | --- | --- | --- |
| 100% whole grains: 3 servings per day |  |  |  |  |  |  |  |  |  |  |  |  |  |  |
| Berries: 2+ servings per week |  |  |  |  |  |  |  |  |  |  |  |  |  |  |
| Dark leafy greens: 6+ servings per week |  |  |  |  |  |  |  |  |  |  |  |  |  |  |
| Other vegetables: 1+ serving per day |  |  |  |  |  |  |  |  |  |  |  |  |  |  |
| Legumes: 3+ servings per week |  |  |  |  |  |  |  |  |  |  |  |  |  |  |
| Nuts: 5+ servings per week |  |  |  |  |  |  |  |  |  |  |  |  |  |  |
| Extra virgin olive oil: primary oil used at home |  |  |  |  |  |  |  |  |  |  |  |  |  |  |
| Yogurt: 0-7 servings per week* |  |  |  |  |  |  |  |  |  |  |  |  |  |  |
| Fish: 1+ serving per week* |  |  |  |  |  |  |  |  |  |  |  |  |  |  |
| Poultry: 0-3 servings per week* |  |  |  |  |  |  |  |  |  |  |  |  |  |  |
| Red meat: 0-2 servings per week* |  |  |  |  |  |  |  |  |  |  |  |  |  |  |
| Egg yolks: 0-4 per week* |  |  |  |  |  |  |  |  |  |  |  |  |  |  |
| Cheese: 0-3 servings per month* |  |  |  |  |  |  |  |  |  |  |  |  |  |  |
| Sweets, candy, ice cream: 0-4 servings per week |  |  |  |  |  |  |  |  |  |  |  |  |  |  |
| Wine: 0-5 ounces per day** |  |  |  |  |  |  |  |  |  |  |  |  |  |  |

*Does not apply if you follow a vegan diet.

**If you do not drink alcohol, do not start.
